# Variation in sleep and metabolic function is associated with latitude and average temperature in *Drosophila melanogaster*

**DOI:** 10.1002/ece3.3963

**Published:** 2018-03-26

**Authors:** Elizabeth B. Brown, Joshua Torres, Ryan A. Bennick, Valerie Rozzo, Arianna Kerbs, Justin R. DiAngelo, Alex C. Keene

**Affiliations:** ^1^ Department of Biological Sciences Florida Atlantic University Jupiter FL USA; ^2^ Wilkes Honors College Florida Atlantic University Jupiter FL USA; ^3^ Division of Science Pennsylvania State University Berks Reading PA USA; ^4^ Lifelong Learning Society Florida Atlantic University Jupiter FL USA; ^5^ Dwyer High School Palm Beach Gardens FL USA

**Keywords:** clinality, evolution, metabolism, natural variation, sleep, starvation resistance

## Abstract

Regulation of sleep and metabolic homeostasis is critical to an animal's survival and under stringent evolutionary pressure. Animals display remarkable diversity in sleep and metabolic phenotypes; however, an understanding of the ecological forces that select for, and maintain, these phenotypic differences remains poorly understood. The fruit fly, *Drosophila melanogaster*, is a powerful model for investigating the genetic regulation of sleep and metabolic function, and screening in inbred fly lines has led to the identification of novel genetic regulators of sleep. Nevertheless, little is known about the contributions of naturally occurring genetic differences to sleep, metabolic phenotypes, and their relationship with geographic or environmental gradients. Here, we quantified sleep and metabolic phenotypes in 24 *D. melanogaster* populations collected from diverse geographic localities. These studies reveal remarkable variation in sleep, starvation resistance, and energy stores. We found that increased sleep duration is associated with proximity to the equator and elevated average annual temperature, suggesting that environmental gradients strongly influence natural variation in sleep. Further, we found variation in metabolic regulation of sleep to be associated with free glucose levels, while starvation resistance associates with glycogen and triglyceride stores. Taken together, these findings reveal robust naturally occurring variation in sleep and metabolic traits in *D. melanogaster*, providing a model to investigate how evolutionary and ecological history modulate these complex traits.

## 1 INTRODUCTION

Species display robust differences in homeostatically regulated behaviors including sleep, feeding, and metabolic function, yet little is known about the ecological and functional relationship between these traits (Aulsebrook, Jones, Rattenborg, Roth, & Lesku, 2016; Eban‐Rothschild, Giardino, & de Lecea, 2017). In mammals, sleep duration ranges from ~2 to 18 hr/day, suggesting that the environment and evolutionary history potently affect sleep regulation (Capellini, Barton, McNamara, Preston, & Nunn, 2008). While the ecological factors that drive differences in sleep remain largely unknown, a central hypothesis is that variation in sleep is linked to an animal's metabolic and foraging needs (Capellini et al., 2008; Siegel 2005). Supporting this notion, variation in sleep duration in *Drosophila* is associated with latitudinal cline (Svetec, Zhao, Saelao, Chiu, & Begun, 2015), raising the possibility that temperature and food availability also impact sleep need. Determining the relationship between sleep and metabolic function, and how evolution shapes these processes, is critical for understanding the function of sleep and basis for sleep differences between and within species.

The fruit fly *Drosophila melanogaster* presents a powerful model for investigating genetic interactions between sleep and metabolic processes (Erion, DiAngelo, Crocker, & Sehgal, 2012; Yurgel, Masek, DiAngelo, & Keene, 2014). High throughput measurements of fly sleep and activity can be obtained using *Drosophila* activity monitors (DAMS), where infrared beam breaks are indicative of fly movement (Pfeiffenberger, Lear, Keegan, & Allada, 2010a). Sleep is measured by 5 min of inactivity because it correlates with all other characteristics of sleep (Shaw, Cirelli, Greenspan, & Tononi, 2000). Flies acutely modulate their sleep in accordance with nutrient availability, and starvation potently inhibits sleep and initiates foraging, thereby providing a system to investigate the relationship between sleep and metabolic regulation (Keene et al., 2010; Lee & Park, 2004; Linford, Chan, & Pletcher, 2012). Genetic evidence suggests sleep and metabolic function are highly conserved from flies to mammals at the molecular, pharmacological, and physiological levels (Allada & Siegel, 2008; Padmanabha & Baker, 2014), indicating that flies are an excellent system to examine the interactions between sleep and metabolic function.

While screens of inbred *Drosophila* lines have identified many regulators of sleep and metabolism (Cirelli et al., 2005; Koh et al., 2008; Rogulja & Young, 2012), naturally occurring variation has also been leveraged to identify the genetic architecture regulating these processes (Harbison, Carbone, et al., 2009; Harbison, McCoy, & Mackay, 2013; Hardy et al., 2018). Quantitative genetic approaches in fully sequenced lines have provided insight into the genetic basis for resistance to environmental and physiological stressors including starvation resistance, and identified novel regulators of sleep (Harbison, Yamamoto, Fanara, Norga, & Mackay, 2004; Vieira et al., 2000). Further, experimental evolution and artificial selection approaches have revealed a relationship between sleep, feeding, and starvation resistance (Masek et al., 2014; Slocumb et al., 2015). For example, selection for short‐sleeping flies results in reduced energy stores and sensitivity to starvation, while selecting for starvation resistance increases sleep duration (Masek et al., 2014; Seugnet et al., 2009). These studies have provided insight into the genetic and functional relationship between sleep and metabolic regulation; yet, the ecological factors that shape the diversity of these traits, such as temperature, lighting, humidity, and distribution of food resources in naturally occurring populations remain poorly understood.

Inspired by previous work investigating the relationship between geographic locality and sleep regulation (Svetec et al., 2015), we examined the relationship between sleep, metabolic function, and environmental localities. Here, we describe the analysis of sleep regulation, starvation resistance, the effects of starvation on sleep, and measurements of nutrient storage in *D. melanogaster* collected from geographically distinct localities that differ in latitude, longitude, temperature, and altitude to determine the environmental and geographic factors that associate with sleep regulation. We tested 24 populations of outbred *D. melanogaster* for these behavioral and physiological variables, providing insight into the relationship between these traits and their association with geographic locality. Our findings reveal highly significant variation in all traits measured, suggesting these traits are influenced by their environmental and evolutionary history.

## 2 METHODS

### 2.1 *Drosophila* maintenance

All populations were obtained from the *Drosophila* Species Stock Center (University of California, San Diego) with stock numbers provided in Table 1. They were maintained at this stock center on standard *Drosophila* media at 18–25°C for between 8 and 63 years. In the laboratory, flies were reared and maintained on a 12:12 light–dark cycle in humidified incubators at 25°C and 65% humidity (Percival Scientific, Perry, IA). Unless otherwise noted, all flies were maintained and tested on a standard cornmeal/agar medium used by the Bloomington *Drosophila* Stock Center, consisting of yeast, soy flour, cornmeal, malt extract, agar, light corn syrup, and propionic acid (http://flystocks.bio.indiana.edu/Fly_Work/media-recipes/bloomfood.htm).

**Table 1:** The localities of each *Drosophila melanogaster* population and their corresponding geographic variables. Variables include latitude, longitude, altitude, and average temperature. For each population, its stock number and date of collection are also listed

Line	Stock	Locality	Collection date	Latitude	Longitude	Altitude	Average temperature
1	14021‐0231.58	Bermuda	1954	32°18′28.08″N	64°45′1.80″W	14.16	23.20
2	14021‐0231.134	American Samoa	2009	14°18′5.90″S	170°41′46.25″W	238.29	26.55
3	14021‐0231.132	Southwest Harbor, Maine	2009	44°16′47.37″N	68°19′29.97″W	11.35	6.58
4	14021‐0231.59	Bogota, Colombia	1962	4°42′39.56″N	74° 4′19.53″W	2,581.03	20.51
5	14021‐0231.69	Athens, Greece	1965	37°59′1.71″N	23°43′39.14″E	71.08	16.95
6	14021‐0231.64	Kariba Dam, Zimbabwe	1963	16°31′19.58″S	28°45′42.01″E	664.14	23.34
7	14021‐0231.131	La Jolla, California	2009	32°49′58.12″N	117°16′16.58″W	51.89	16.49
8	14021‐0231.136	Fukushima, Japan	2009	37°45′39.00″N	140°28′29.02″E	68.24	11.72
9	14021‐0231.67	Pyrenees, Spain	1965	42°40′5.45″N	1° 0′4.28″E	2,314.12	8.43
10	14021‐0231.129	Cebu, Philippines	2008	10°18′56.52″N	123°53′7.57″E	56.31	27.19
11	14021‐0231.137	Ogasawara Islands, Japan	2009	27° 4′30.19″N	142°12′41.77″E	223.45	24.38
12	14021‐0231.61	Blacksburg, Virginia	1954	37°13′46.46″N	80°24′50.18″W	634.67	11.58
13	14021‐0231.23	Crete, Greece	2002	35°14′24.42″N	24°48′33.37″E	1,586.60	18.30
14	14021‐0231.03	Queensland, Australia	Not Listed	20°55′3.27″S	142°42′10.06″E	321.11	25.09
15	14021‐0231.43	San Luis Potosi, Mexico	2005	22° 9′23.29″N	100°59′7.95″W	1,877.06	18.85
16	14021‐0231.15	Bahia, Brazil	Not Listed	12°34′47.06″S	41°42′2.62″W	715.73	22.13
17	14021‐0231.130	Queensferry, Scotland	2009	55°59′24.01″N	3°23′56.56″W	19.71	7.45
18	14021‐0231.22	Chiapas, Mexico	2002	16°45′24.95″N	93° 7′45.25″W	549.63	24.23
19	14021‐0231.01	Ica, Peru	1956	14° 4′31.66″S	75°44′3.05″W	399.25	19.25
20	14021‐0231.35	Monkey Hill, St. Kitts	2005	17°19′26.30″N	62°43′30.14″W	120.42	27.24
21	14021‐0231.56	Plainville, Connecticut	2007	41°40′32.68″N	72°51′48.11″W	52.86	9.74
22	14021‐0231.62	Cape Town, South Africa	1954	33°55′29.53″S	18°25′26.60″E	52.57	16.35
23	14021‐0231.68	Israel	1954	31° 2′45.78″N	34°51′5.80″E	287.34	19.93
24	14021‐0231.66	Madeira, Portugal	1965	32°45′38.55″N	16°57′34.10″W	1,581.31	19.73

### 2.2 Behavioral analysis

Upon eclosion, male and flies were transferred to empty vials containing standard cornmeal/agar medium for 24–48 hr to age and mate. At 3–5 days of age, flies were then briefly anesthetized using CO_2_, females were sorted and then individually placed into plastic tubes containing standard food media for behavioral monitoring. Flies were then acclimated to these conditions for at least 24 hr prior to testing. Fly activity was monitored using DAM2 DAMS (Trikinetics, Waltham, MA) as previously described (Hendricks et al., 2000; Shaw et al., 2000). The DAM system measures activity by monitoring the number of infrared beam crossings for each fly. These data were then used to calculate sleep‐related traits by extracting immobility bouts of 5 min or more using the *Drosophila* Sleep Counting Macro (Pfeiffenberger, Lear, Keegan, & Allada, 2010b). Multiple variables of sleep were analyzed, including total sleep duration, waking activity, sleep bout number, and average sleep bout length as previously described (Pfeiffenberger et al., 2010b; Pitman, McGill, Keegan, & Allada, 2006). For experiments examining the effects of starvation on sleep, activity was recorded for 1 day on standard food media prior to transferring flies into tubes containing 1% agar (Fisher Scientific, Hampton, NH) at the start of lights on at zeitgeber time (ZT) 0. To calculate starvation‐induced sleep suppression, the within‐fly percentage change in sleep was calculated as follows: ([% sleep starved − % sleep baseline]/[% sleep baseline]) × 100 (Keene et al., 2010; Murakami et al., 2016). This calculation accounts for differences in baseline sleep and accurately reflects the response to starvation. The same flies used to measure sleep and starvation induced sleep suppression were also used in measurements of starvation resistance, which was assessed once flies were transferred to tubes containing 1% agar. The time of death was manually determined as the very last bout of waking activity for each individual fly.

### 2.3 Climate data

Latitude, longitude, and altitude measurements for each locality were obtained from Google Earth (https://www.google.com/earth/). Latitude measurements were adjusted to represent the absolute distance from the equator. Longitude measurements to the east of the Prime Meridian were assigned a positive value, while measurements to the west were assigned a negative value. Altitude measurements were obtained from the latitudinal and longitudinal coordinates of each locality. As these measurements were not normally distributed, the Log_10_ of altitude was used. Temperature data were obtained from Berkeley Earth (http://berkeleyearth.org/). Monthly temperature measurements were obtained from a 1° × 1° latitude‐longitude grid covering surface of the Earth. Average temperature was calculated as the average of the monthly temperature measurements.

### 2.4 Triglyceride, glucose, and glycogen measurements

Whole flies, aged 3–5 days, were collected and flash‐frozen on dry ice at ZT 0 for subsequent analyses. Protein, glucose, glycogen, and triglyceride measurements were then performed as previously described (Gingras, Warren, Nagengast, & Diangelo, 2014; Sassu et al., 2012). Briefly, two headless female flies aged 3–5 days were homogenized in 50 mmol/L Tris‐HCl, pH 7.4, 140 mmol/L NaCl, 0.1% Triton‐X, and 1× protease inhibitor cocktail (Sigma Aldrich, St Louis, MO). Triglyceride levels were measured using the Infinity Triglycerides Kit (Fisher Scientific, Hampton, NH), while protein levels were measured using the Pierce BCA Protein Assay Kit (Fisher Scientific, Hampton, NH). Total glucose levels were determined using the Glucose Oxidase Reagent (Pointe Scientific, Canton, MI) in samples previously treated with 8 mg/mL amyloglucosidase in 0.2 mol/L Sodium Citrate buffer, pH 5.0 (Boston BioProducts, Ashland, MA) for 2 hr. Glycogen levels were determined by measuring free glucose in samples not treated with amyloglucosidase and then subtracting the free glucose from total glucose concentration. For each sample, triglyceride, glycogen, and free glucose levels were standardized to the total protein content.

### 2.5 Statistics

First, to assess the normality of each trait for each population, we performed a Shapiro*–*Wilk test. For several traits, there was at least one non‐normally distributed population, including: waking activity, bout length, % change in sleep, and measurements of glycogen levels. To assess variation among populations in traits where not all populations were normally distributed, we performed the nonparametric Wilcoxon rank‐sum test. To assess variation among populations in traits in which all populations were normally distributed, we performed a one‐way analysis of variance (ANOVA): *Y* = μ + Line + ε, where Line represents the fixed effect of population from a given locality and ε indicates error. In order to determine whether a given population differed from the global average, we performed a one‐sample *t*‐test and then adjusted for multiple testing using Bonferroni's correction. A one‐sample *t*‐test was also performed to determine whether sleep responses as a result of starvation were significantly different from zero. Differences in survival upon starvation were assessed using the nonparametric log‐rank test. Linear regressions were used to determine the relationship between a given trait and geographic variable as well as between two traits. For each regression analysis, with the exception of measurements of starvation resistance, the population average of each trait was used. For starvation resistance, the median time until death (LT50) was used. To account for multiple testing, Bonferroni correction was performed for each correlation between a given continental unit and geographic variable. All data were analyzed using JMP 12.0 software (SAS Institute Inc., Cary, NC).

## 3 RESULTS

**Figure 1:**
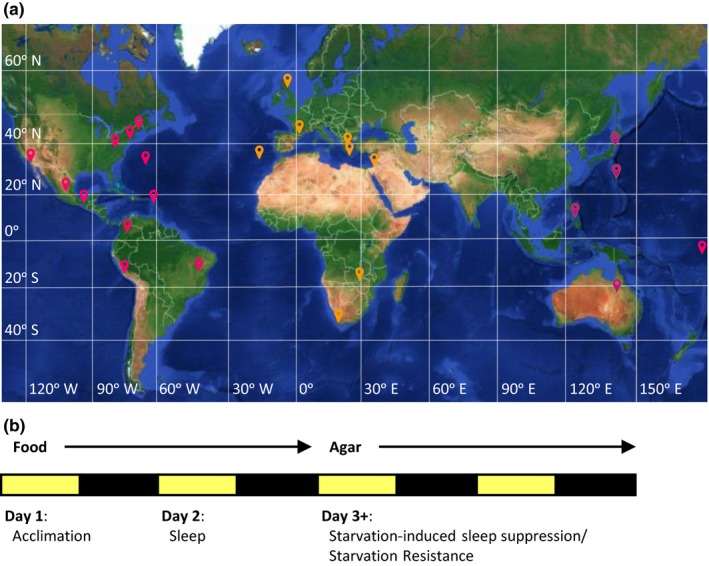
Localities of *Drosophila melanogaster* populations and experimental design. (a) World map displaying the localities (red pins) of each the 24 populations. Horizontal lines indicate latitude and vertical lines indicate longitude. (b) Schematic of experimental protocol used to assess sleep‐ and metabolic‐related traits

To determine the contribution of geographic variation on sleep and metabolic regulation, we obtained 24 populations of *D. melanogaster* from diverse localities throughout the world (Figure 1a; Table 1). To assess differences in sleep and metabolic function within individual flies, sleep was measured in 3‐ to 5‐day‐old female flies on food. Following 24 hr of sleep acquisition, flies were transferred to starvation tubes containing agar alone and maintained on this substrate to measure starvation‐induced changes in sleep and starvation resistance (Figure 1b), providing multiple metrics of sleep and metabolic function within an individual animal.

### 3.1 Variation in sleep traits

**Figure 2:**
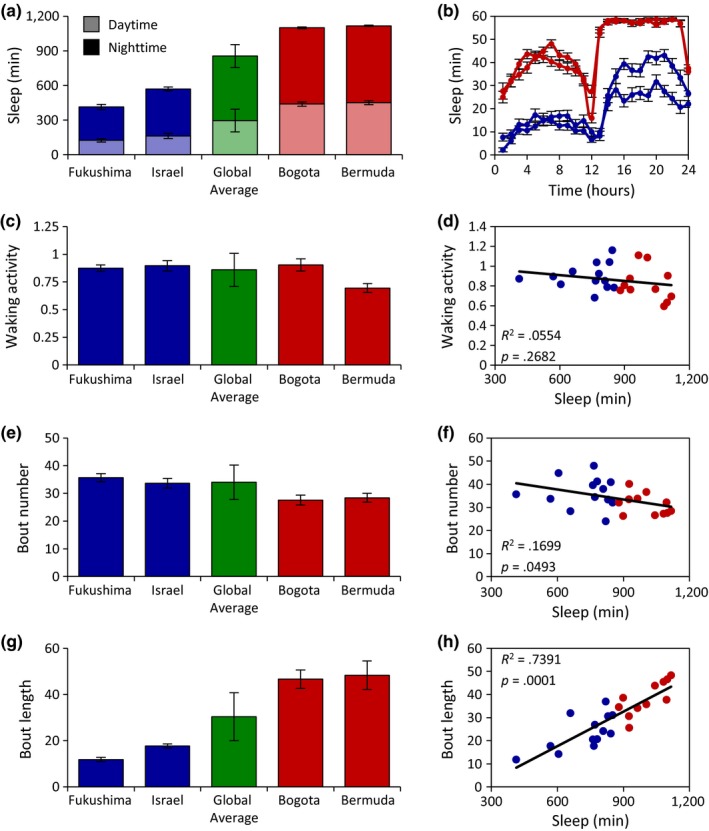
Measurements of sleep‐related traits reveal significant variation among *Drosophila melanogaster* populations. (a) Daytime (light bars) and nighttime (dark bars) sleep duration for the two shortest and longest sleeping populations relative to the global average. There is significant variation in both daytime and nighttime sleep duration among the populations tested (daytime: ANOVA:* F*_23,811_ = 23.2805, *p *<* *.0001; nighttime: ANOVA:* F*_23,811_ = 39.2778, *p *<* *.0001). (b) Sleep profiles over a 24‐hr period of the two shortest and longest sleeping populations. (c) Measurements of waking activity for the two shortest and longest sleeping populations relative to the global average. There is significant variation in waking activity among the populations tested (Wilcoxon rank‐sum test: χ^2^ = 240.7045, *p *<* *.0001). (d) There is no correlation between sleep duration and waking activity. (e) Measurements of bout number for the two shortest and longest sleeping populations relative to the global average. There is significant variation in bout number among the populations tested (ANOVA:* F*_23,811_ = 11.7775, *p *<* *.0001). (f) There is a moderate, but significant correlation between sleep duration and bout number. (g) Measurements of bout length for the two shortest and longest sleeping populations relative to the global average. There is significant variation in bout length (Wilcoxon rank‐sum test: χ^2^ = 340.777, *p *<* *.0001) among the populations tested. (h) There is a significant correlation between sleep duration and bout length. For each population, data shown are mean ± *SE*. For global averages, data shown are mean ± *SD*. Blue dots represent populations whose sleep duration is below the global average, while red dots represent populations above the global average. For measurements of sleep‐related traits for all localities, see Table 2

Quantification of sleep duration in fed flies revealed remarkable diversity in sleep duration and architecture between fly populations (Figure 2; Table 2). The average sleep duration of all 24 lines tested for 24 hr on food was 855 min. The shortest sleeping populations include flies from Fukushima, Japan, and Israel, sleeping on average 413 and 570 min, respectively (Figure 2a; Table 2). In addition, several long‐sleeping populations were identified, including flies from Bogota, Columbia (1,100 min), and Bermuda (1,117 min) (Figure 2a, Table 2). The differences between short‐ and long‐sleeping flies were present during both the day and night, thereby suggesting that the observed phenotypes are not the result of altered circadian regulation (Figure 2b).

**Table 2:** Mean responses of measurements of sleep and starvation resistance for each locality. The standard error (*SE*) is shown to the right of each mean, while the sample sizes are depicted on the far right

Locality	Sleep duration		Waking activity		Bout number		Bout length		% Change in sleep		Starvation resistance	Sample size
	Average	*SE*	Average	*SE*	Average	*SE*	Average	*SE*	Average	*SE*	LT50	
Bermuda	1,117.22	19.08	0.69	0.04	28.42	1.63	48.31	6.17	−12.33	2.43	1.86	32
American Samoa	1,083.00	18.95	0.59	0.04	27.26	1.50	45.50	3.65	−11.58	2.13	3.44	34
Southwest Harbor, Maine	660.25	29.46	0.94	0.03	28.30	1.72	31.92	5.77	−6.22	7.23	3.87	40
Bogota, Colombia	1,100.31	23.85	0.90	0.06	27.59	1.80	46.61	3.96	−8.95	1.95	2.62	40
Athens, Greece	842.90	33.53	1.16	0.08	40.90	2.13	23.10	1.87	−32.90	7.31	2.02	33
Kariba Dam, Zimbabwe	1,096.35	22.06	0.63	0.04	32.11	1.41	37.77	2.60	−41.64	5.64	2.44	39
La Jolla, California	771.00	44.35	1.04	0.05	34.47	2.10	26.95	3.77	5.03	5.30	3.69	36
Fukushima, Japan	413.08	30.36	0.87	0.03	35.64	1.46	11.80	0.95	−28.38	7.95	3.00	32
Pyrenees, Spain	781.58	41.04	0.92	0.04	41.24	1.66	20.64	1.81	−24.64	6.53	3.07	40
Cebu, Philippines	927.34	31.24	0.76	0.02	40.09	1.76	25.61	2.15	1.22	3.53	4.01	36
Ogasawara Islands, Japan	1,043.28	21.12	0.77	0.03	26.59	1.40	43.79	3.08	−14.28	2.14	3.90	32
Blacksburg, Virginia	830.90	35.61	1.04	0.06	33.36	2.08	30.58	2.96	1.67	5.30	2.61	40
Crete, Greece	964.58	30.65	1.11	0.06	33.75	1.93	34.06	3.42	−14.19	3.51	3.67	40
Queensland, Australia	762.12	24.85	0.68	0.03	39.61	1.77	20.56	1.13	−19.29	6.51	1.94	32
San Luis Potosi, Mexico	809.13	31.61	0.85	0.03	37.88	1.74	24.14	1.87	−7.23	5.43	3.85	40
Bahia, Brazil	880.31	33.90	0.75	0.02	32.00	1.92	34.56	4.55	−0.90	5.75	3.68	32
Queensferry, Scotland	925.31	33.84	0.87	0.05	33.47	1.54	30.58	2.50	−19.66	3.99	3.59	32
Chiapas, Mexico	820.32	25.32	0.79	0.03	23.94	1.40	36.98	1.89	−8.37	2.66	3.23	32
Ica, Peru	766.61	35.14	0.85	0.04	47.94	2.28	17.78	1.56	−36.79	4.30	6.00	32
Monkey Hill, St. Kitts	899.08	19.76	0.81	0.02	26.21	1.28	38.56	2.73	0.05	3.30	3.72	32
Plainville, Connecticut	851.25	23.76	0.78	0.03	32.03	1.82	31.05	2.69	9.99	4.16	3.29	32
Cape Town, South Africa	1,005.29	39.30	1.09	0.06	36.56	2.45	35.76	4.10	−43.59	4.82	1.97	39
Israel	569.86	29.09	0.90	0.05	33.66	1.72	17.69	0.90	6.80	5.60	2.97	32
Madeira, Portugal	605.56	33.41	0.82	0.04	44.78	1.80	14.29	1.14	39.31	11.79	3.90	40

Given that these populations have been stored in laboratory conditions for between 8 and 63 years, we next assessed whether the duration of time spent in these conditions may have influenced the extensive variation in sleep duration we observed. We found no association between the date a given population was collected and either the average sleep duration or the coefficient of variation of sleep duration (Figure S1), suggesting that variation in sleep regulation across populations is not associated with the length of time animals were housed in laboratory. It is also possible that differences in sleep duration are reflective of lethargy or hyperactivity rather than sleep per se. To investigate this, we measured the relationship between sleep duration and waking activity. We observed no difference in waking activity between the two shortest sleeping populations and the global average, while a moderate reduction was observed in one of the long sleeping populations (Figure 2c). A regression analysis of the 24 populations tested revealed a lack of correlation between sleep duration and waking activity (Figure 2d), suggesting that these traits are independently regulated and that the differences in sleep across geographic localities are not the result of hyperactivity or lethargy.

Sleep duration is composed of individual sleep bouts and may be enhanced by increasing the number of bouts or the length of each individual sleep bout within a given day (Pfeiffenberger et al., 2010b). To differentiate between these possibilities, we calculated the average number of bouts for each population. Overall, the average number of bouts ranged from 24 to 48 per 24 hr (Figure 2e; Table 2), and there was a moderate but significant correlation between bout number and sleep duration (Figure 2f), suggesting that sleep initiation only mildly associates with variation in sleep duration. In addition, the length of individual sleep bouts was shorter in the Fukushima, Japan and Israel populations, and longer in the Bogota, Columbia and Bermuda populations compared to the global average (Figure 2g). Furthermore, a regression analysis revealed a highly significant correlation between sleep duration and bout length (Figure 2h). Taken together, these findings suggest that the diversity in sleep duration is primarily conferred by increasing the length of individual sleep bouts.

**Figure 3:**
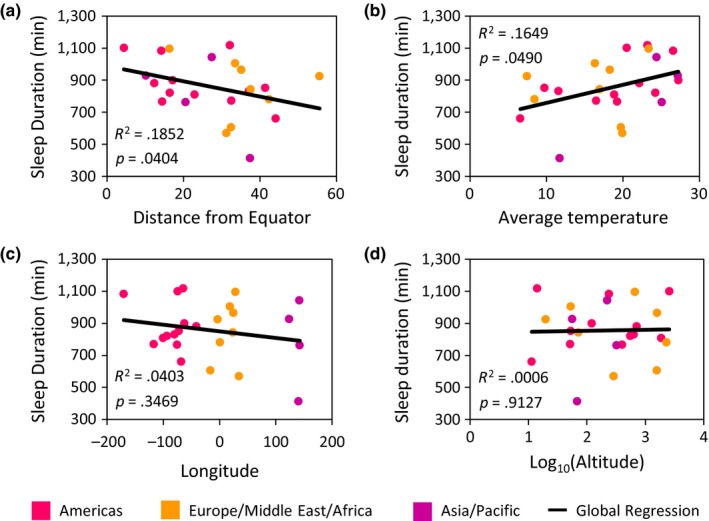
Linear regression analyses highlight the relationship between sleep duration and geographic variables. There is a significant relationship between (a) sleep duration and distance from the equator as well as (b) sleep duration and temperature. There is no relationship between (c) sleep duration and longitude nor between (d) sleep duration and altitude. Each population is color coded to represent their respective continental unit. Statistical analyses are reported in Table 2

A previous study examining *D. melanogaster* collected from five North American localities suggested that sleep is increased in equatorial regions (Svetec et al., 2015). To determine how sleep and activity relate to variation in geographic and environmental locality, we performed linear regressions on four geographic variables including latitude, longitude, altitude, and temperature. This analysis revealed an association between sleep duration and both latitude and temperature, while no relationship was observed between sleep duration and longitude or altitude (Figure 3; Appendix S1). When divided into continental units, we observed a significant negative correlation between sleep duration and temperature in Asia/Pacific as well as the Americas, while no correlation was observed between populations originating from Europe/Middle East/Africa (Figure 3a). We also observed a significant positive correlation between waking activity and temperature (Appendix S1). Although this association includes all localities tested, this trend is most strongly evident in populations from the Americas. Taken together, these results suggest that close proximity to the equator and increased temperature are both associated with increased sleep duration and reduced waking activity.

### 3.2 Variation in the metabolic regulation of sleep

**Figure 4:**
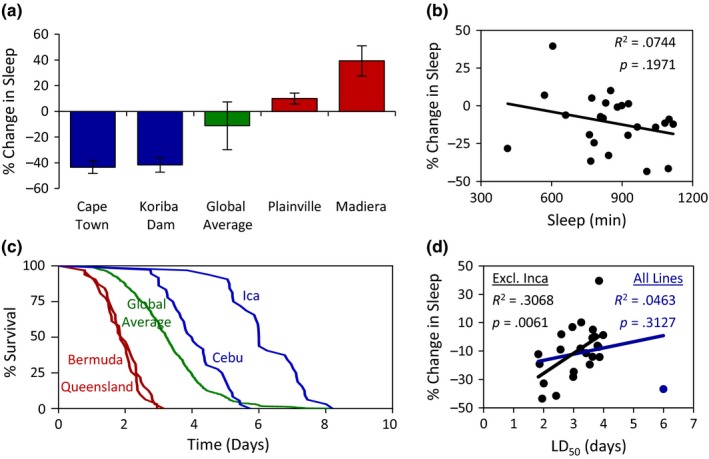
The effect of starvation on sleep and longevity in *Drosophila melanogaster* populations. (a) There is significant variation in starvation‐induced changes in sleep duration (Wilcoxon rank‐sum test: χ^2^ = 185.9639, *p *<* *.0001). The change in sleep for the two shortest and longest sleeping populations is shown relative to the global average. For each population, data shown are mean ± *SE*. For global average, data shown are mean ± *SD*. (b) There is no relationship between sleep duration and starvation‐induced changes in sleep. (c) There is significant variation in measurements of starvation resistance (Log‐rank test: χ^2^ = 811.1161, *p *<* *.0001). Survivorship curves for the two least and most starvation resistant populations are shown relative to the global average. (d) The relationship between starvation resistance and starvation‐induced changes in sleep. The blue regression line includes Ica, Peru, the outlier population (blue dot), while the black regression lines do not. For measurements of starvation‐induced sleep suppression and starvation resistance for all localities, see Table 2

Flies, like mammals, suppress sleep when starved, presumably to initiate a foraging response (Danguir & Nicolaidis, 1979; Keene et al., 2010). This phenotype has been extensively investigated in inbred fly lines; however, it has not been studied in outbred populations of *Drosophila* (Yurgel et al., 2014). To determine how sleep is modulated by nutrient deprivation, flies were starved following the 24‐hr sleep recordings on food by being transferred to tubes containing agar alone and the change in sleep between the two housing conditions was then determined. After 24 hr of starvation, twelve lines significantly suppressed sleep in response to starvation, eleven lines exhibited no change in sleep, and a single line significantly increased sleep (Appendix S2). Further, after 48 hr, the number of lines that significantly suppressed sleep in response to starvation increased to twenty. Flies from Cape Town, South Africa (44%) and Kariba Dam, Zimbabwe (42%) displayed the greatest sleep suppression, while flies Madeira, Spain (39%) showed the greatest increase (Figure 4a). There was no correlation between sleep duration on food and starvation‐induced sleep suppression (Figure 4b), suggesting that sleep duration and changes in sleep resulting from starvation are independently regulated.

It is possible that sleep suppression in response to starvation represents a specific sensitivity to sleep regulation or provides a more general indicator of starvation tolerance. To measure starvation resistance, we maintained flies on agar in activity monitors and measured time of death. The mean starvation resistance for all 24 lines tested was 3.26 days. The least resistant populations include flies from Bermuda and Queensland, Australia, living on average 1.86 and 1.93 days, respectively. The most resistant populations include flies from Cebu, Philippines, living 4.01 days and Ica, Peru, living 6.00 days (Figure 4c). We observed no correlation between starvation‐induced sleep suppression and starvation resistance when all flies were included in the regression (blue line; Figure 4d). Given that flies from Ica, Peru live approximately 2 days longer than the next longest‐living population and over three standard deviations above the global average, we next performed a linear regression in the absence of this outlier locality. We found a strong positive correlation between starvation‐induced sleep suppression and starvation resistance (black line: Figure 4d), suggesting that sleep duration and changes in sleep resulting from starvation are not independently regulated. To determine whether starvation‐induced sleep suppression and/or starvation resistance is associated with variation in geographic variables, we performed linear regression analyses between these traits and the geographic variables. For both traits, we did not observe any correlation with the geographic variables measured (Appendix S3). Taken together, these findings demonstrate dramatic variation in starvation resistance and the metabolic regulation of sleep in different fly populations, indicating that the metabolic regulation of sleep is coregulated with starvation resistance.

### 3.3 Variation in nutrient storage

**Figure 5:**
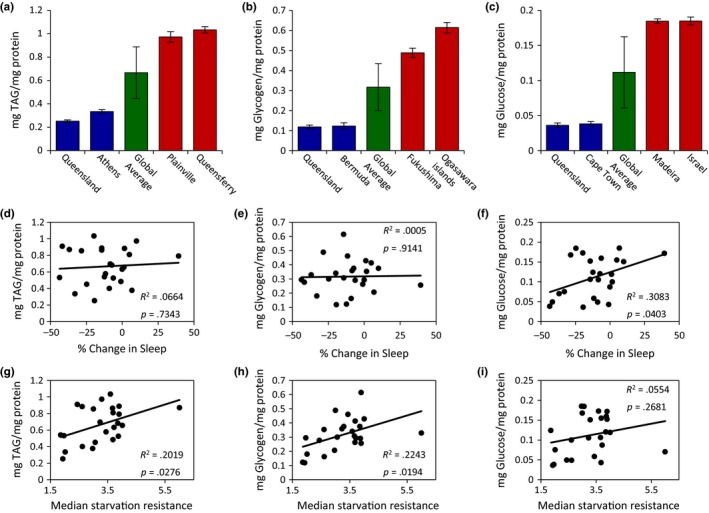
Energy storage measurements of *Drosophila melanogaster* populations. (a) There is significant variation in triglyceride (TAG) levels (a; ANOVA:* F*_23,309_ = 34.8198, *p *<* *.0001), glycogen (b; Wilcoxon rank‐sum test: χ^2^ = 202.5611, *p *<* *.0001), and free glucose levels (c; ANOVA:* F*_23,310_ = 79.1969, *p *<* *.0001). For each trait, the two populations with the lowest and highest measurements are shown relative to the global average. For each population, data shown are mean ± *SE*. For global average, data shown are mean ± *SD*. (d–f) The relationships between starvation‐induced sleep suppression and energy storage measurements. There is no correlation between starvation‐induced sleep suppression and (d) triglyceride or (e) glycogen levels. However, starvation‐induced sleep suppression was significantly correlated with (f) free glucose levels. (g–i) The relationships between starvation resistance and energy storage measurements. There is a significant correlation between starvation resistance and (g) triglyceride and (h) glycogen levels, while there is no correlation between starvation resistance and (i) free glucose levels. For measurements of nutrient stores for all localities, see Appendix S4

Energy stores and circulating nutrients potently affect both sleep and starvation resistance. To investigate the relationship between energy stores and these processes, we measured triglyceride levels, glycogen levels, and free glucose across all 24 populations. We identified at least a fourfold difference in triglyceride, glycogen, and free glucose levels in the populations tested (Figure 5a–c; Appendix S4). Across all three measurements, energy stores were lowest in the Queensland, Australia population. A linear regression analysis of the relationship between energy levels and starvation‐induced sleep suppression revealed no significant correlation between starvation‐induced changes in sleep and triglyceride or glycogen levels (Figure 5d,e). However, a significant correlation was observed between starvation‐induced regulation of sleep and free glucose, suggesting that flies with lower levels of free glucose during the fed state display a greater reduction in sleep upon starvation (Figure 5f). Conversely, increased starvation resistance correlated with elevated levels of triglycerides and glycogen, but not free glucose (Figure 5g–i), indicating elevated energy stores in fed flies are indicators of starvation resistance. Taken together, these findings suggest that energy storage molecules (glycogen and triglycerides) and free glucose have distinct effects on the metabolic regulation of sleep and starvation resistance.

**Figure 6:**
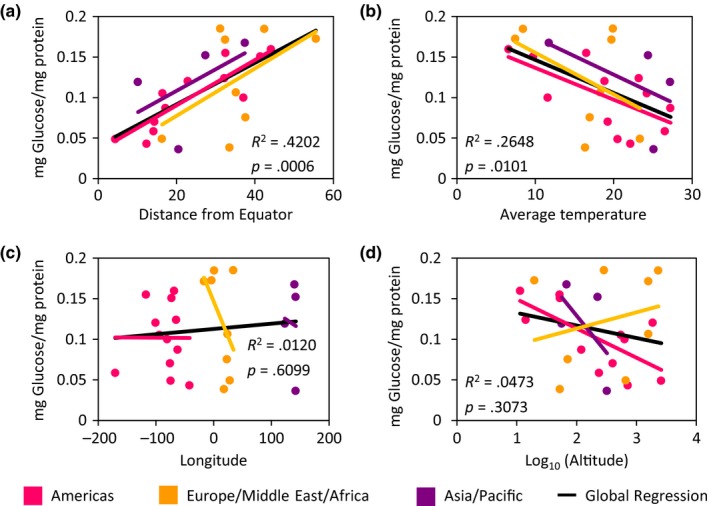
Linear regression analyses highlight the relationship between free glucose and geographic variables. There is a significant positive relationship between (a) free glucose levels and distance from the equator, while there is a significant negative relationship between (b) free glucose and temperature. There is no relationship between free (c) glucose levels and longitude nor between (d) free glucose and altitude. Each population is color coded to represent their respective continental unit. Statistical analyses are reported in Appendix S5

To determine the relationship between energy stores and free glucose with geographic variables, we again performed linear regression analyses between these traits and the geographic variables. These analyses revealed no correlation between triglyceride or glycogen levels and any geographic variables measured (Appendix S5). However, we did observe a correlation between free glucose levels and two of the geographic variables (Figure 6). We found that free glucose levels were significantly correlated with increased distance from the equator and decreased mean annual temperatures, and that these associations are strongest in populations from the Americas (Figure 6a,b; Appendix S5). Therefore, variation in both latitude and temperature appear to associate with the regulation of sleep duration and free glucose.

## 4 DISCUSSION

The expansive radiation of *D. melanogaster* provides an excellent opportunity to examine the interrelationship between natural variation in complex behaviors, physiological traits, and the environmental factors that may act as selective forces to shape such variation. Here we examined natural variation in sleep and metabolic function, as well as their relationship with each other, and with several geographic and environmental gradients, by investigating 24 *D. melanogaster* populations from localities across the globe. Our assessment of sleep and metabolic traits over a wide geographic range has made it possible to identify general trends in sleep‐related traits, as well as evaluate their degree of variation. The vast majority of sleep studies in *Drosophila* have used inbred or isogenic *D. melanogaster* strains. Inbred populations of laboratory *Drosophila* strains typically sleep ~600–800 min daily (Zimmerman, Chan, Jackson, Maislin, & Pack, 2012), while the sleep duration for strains in this study ranged from 413 to 1,117 min. Similarly, starvation resistance in inbred strains is ~36–49 hr, while flies in this study survived up to 144 hr (Gáliková et al., 2015; Mattaliano, Montana, Parisky, Littleton, & Griffith, 2007). Therefore, naturally occurring variability encompasses the sleep and starvation resistant phenotypes that extend well beyond the phenotypes of the commonly used laboratory strains. This remarkable variability in natural populations of *Drosophila* is suggestive of maintenance of genetic and phenotypic variation associated with geographically independent populations of *Drosophila* that persists through years or decades of maintenance. The extreme phenotypes observed in natural populations of *Drosophila* suggest differences between populations could be used to uncover novel genetic architecture associated with natural variation in sleep and metabolic function.

Sleep is regulated by complex genetic architecture and is highly influenced by genetic variation (Cirelli, 2009; Wu, Kumar, Serrano Negron, & Harbison, 2017). While many genes have been identified using mutagenesis approaches, much less is known about the modulation of sleep via naturally occurring genetic variation. For most humans, sleep need is estimated to range from 7 to 9 hr, and single alleles have been identified that robustly influence sleep, a number of which are conserved in *Drosophila* (Allebrandt et al., 2013; He et al., 2009; Shi, Wu, Ptáček, & Fu, 2017). Genome‐wide association studies have identified loci associated with sleep variability, but assessing the contributions of loci to sleep variation is difficult (Kalmbach et al., 2017). Sequenced inbred *Drosophila* lines derived from a wild‐caught population have been previously characterized for variation in sleep, revealing numerous differentially expressed genes and molecular polymorphisms that associate with sleep duration and architecture (Harbison, Mackay, & Anholt, 2009; Harbison et al., 2013). The identification of differences in sleep and metabolic phenotypes from geographically diverse regions presents a complementary approach to investigate the genetic architecture underlying variation in sleep regulation.

Clinal variation has been observed in diverse traits including body size, fecundity, and temperature resistance, but much less is known about the relationship between behavior and clinality [for review, see Adrion, Hahn, and Cooper (2015)]. Here, we observed a moderate association between distance from the equator and sleep duration; however, this was below the threshold of significance after a Bonferroni correction was applied. We also identified a relationship between sleep duration and average temperature. Specifically, we found that increased sleep duration is associated with increased average temperature in Asia/Pacific and the Americas, suggesting that sleep duration is a convergent adaptation to temperature. We did not observe an association with temperature in Europe/Middle East/Africa. It is possible that clinal variation in sleep duration is restricted to specific geographic regions. However, given the limited number of populations within each continental unit (four in the case of Asia/Pacific), we have limited ability to discern this in the present study. Given that there are several temperature‐associated metrics that contribute to the average yearly temperature, including daily/monthly temperature, minimum/maximum temperature, as well as temperature range, another possibility is that a combination of such variables may more robustly associate with variation in sleep duration. Furthermore, the continental units allocated cover vast transects of geography, making it possible that more geographically restricted clines will shed additional light on these geographic associations with sleep duration. These findings are for the most part of modest effect, which may in part be due to the duration of time several of the lines have spent in the laboratory or the lack of sampling depth at any particular locality. Despite these caveats, our worldwide sampling scheme revealed geographic‐scale trend in sleep and metabolic traits.

A previous study using five North American populations found clinal variation in nighttime bout length and sunrise anticipation (Svetec et al., 2015). The moderate clinal relationship between latitude and sleep was observed during the nighttime only, suggesting that ecological variables have differential effects on daytime and nighttime sleep (Svetec et al., 2015). Similarly, we observed no relationship between latitude and daytime sleep, further supporting the notion that clinal variation in sleep duration is nighttime specific. In contrast to Svetec et al., 2015; our analyses include not only sleep behavior, but also traits associated with the metabolic regulation of sleep. Additionally, our analyses take a more global approach to assess variation in sleep and metabolic traits by testing populations from multiple continents. Together, these analyses provide the framework for a more detailed investigation of behavioral adaptations to environmental gradients. Unsurprisingly, there are many additional traits associated with latitudinal clines. Sunrise anticipation, where flies become active prior to the onset of the light cycle, was also associated with latitude (Svetec et al., 2015). Morning anticipation, sleep duration, and temperature‐dependent modulation of sleep are regulated by the circadian clock in *Drosophila* (Agosto et al., 2008; Guo et al., 2016; Parisky, Agosto Rivera, Donelson, Kotecha, & Griffith, 2016). Therefore, it is possible that alterations in circadian neural circuitry or the molecular machinery governing the circadian clock may contribute to cline‐associated differences in sleep among *Drosophila* populations.

Flies robustly suppress sleep and increase activity in response to starvation, and this is mediated by both chemosensory and hormonal factors (Keene et al., 2010; Lee & Park, 2004; Linford et al., 2012; Murakami et al., 2016). Although we did not observe an association between sleep and starvation‐induced sleep suppression, different genetic factors have been previously implicated in these traits (Keene et al., 2010; Yurgel, Masek, DiAngelo, & Keene, 2015). While this phenotype has been reported in diverse genetic backgrounds of *D. melanogaster* (McDonald & Keene, 2010), we found that 10 of the 24 populations tested do not suppress sleep when starved for 24 hr. It is possible that this is associated with a delayed response to starvation. As such, an analysis of sleep during later periods of starvation (48 hr) identified starvation‐induced sleep suppression in eight additional populations. This is further supported by the correlation between starvation induced sleep suppression and starvation resistance. Surprisingly, we identified a single population that significantly increased sleep during starvation, revealing opposing responses to starvation. Although enhanced sleep during starvation has not been reported in *Drosophila*, we have previously identified increased sleep during starvation in the Mexican blind cavefish, and many animals enter hibernation during winter seasons when food availability is scarce (Jaggard et al., 2017; Schmidt, 2014). Therefore, there are likely multiple strategies that are implemented in response to food shortage, including induction of foraging behavior and consequently sleep suppression, or increasing sleep to conserve energy. A better understanding of how environmental factors shape the evolution of these opposing strategies is of particular interest.

In this study, we did not find an association between starvation resistance and starvation‐induced sleep suppression with any environmental gradient, raising the possibility that (1) variation in these traits may be due to an environmental gradient not measured in this study or (2) these traits do not correlate with clinal/geographic variables. Nevertheless, in the case of starvation resistance, several previous studies have also failed to find a relationship with latitudinal cline (Goenaga, Jose Fanara, & Hasson, 2010; Hoffmann, Shirriffs, & Scott, 2005; Robinson, Zwaan, & Partridge, 2000), while others reported negative latitudinal clines (Arthur, Weeks, & Sgrò, 2008; Karan & Parkash, 1998; Karan et al., 1998). In the case of starvation‐induced sleep suppression, the absence of an association with an environmental gradient persisted even when flies were starved for 48 hr. To our knowledge, no similar investigation has been previously performed on the relationship between environmental gradients and starvation‐induced sleep suppression.

Although we did not identify clinal variation in starvation resistance or starvation‐induced sleep suppression, our analysis does not take into account additional variables such as ultraviolet light intensity, seasonality, day length, or other factors that may potently affect food availability and influence selection. It is also possible that there are environmental factors specific to individual localities that shape the behavioral and metabolic responses we observed. One population in which this may indeed be the case is *Drosophila* from Ica, Peru, the most starvation resistant population in our analysis. The city of Ica borders the Atacama Desert, which is classified as a hot desert climate according to the Köppen‐Geiger climate classification system (Peel, Finlayson, & McMahon, 2007). Therefore, it would be informative to assess starvation resistance in additional populations from this region, as well as from other desert climates. Given that this line was collected in 1956, investigating the behavior and physiology of wild‐caught flies from this region, as well as flies from nearby more humid regions, may be informative. We posit that the arid climate of this region would select for *Drosophila* with an increased resistance to starvation, as this is the case with several *Drosophila* species that are found in more xeric habitats (Matzkin & Markow, 2009). Nevertheless, our finding that average temperature is associated with differences in sleep duration and activity across continents suggests a relationship between geographic conditions and sleep regulation.

Upon investigation into the relationship between sleep regulation and energy stores, we found that populations with lower levels of free glucose display a greater increase in sleep suppression after starvation. Limiting glucose utilization pharmacologically suppresses sleep, presumably by mimicking the starvation state (Murakami et al., 2016). These findings suggest natural variation in free glucose associate with how flies modulate sleep in accordance with nutrient shortage. We also found a positive correlation between energy storage measurements (glycogen and triglyceride levels) and starvation resistance. Previous work has shown that artificial selection for increased starvation resistance results in a correlated increase in energy stores (Slocumb et al., 2015), raising the possibility that increased energy storage is an adaptation to areas with limited or sporadic food availability. Of these metabolic traits, we found that free glucose is associated with both equatorial proximity and average temperature, and that clinal variation in populations from the Americas primarily drives this global pattern. Interestingly, allozymes of glucose‐6‐phosphate dehydrogenase (G6PD), an enzyme involved in the breakdown of glucose, also display latitudinal clinality, such that the allozyme with low activity is found at higher frequencies at higher latitudes (Bubliy, Kalabushkin, & Imasheva, 1999; Oakeshott, Chambers, Gibson, Eanes, & Willcocks, 1983; Singh, Hickey, & David, 1982). We can speculate that this may contribute to the higher levels of free glucose; however, functional tests remain an important next step. Overall, this suggests that geographic and environmental gradients are among the selective forces that mediate genetic variation in glucose metabolism and its impact on behavior.

Clinal patterns have been found for numerous phenotypes and their underlying genetic architecture, including allozyme variants, sequence variants, and chromosome inversions (Fabian et al., 2012; Knibb, 1982; Kolaczkowski, Kern, Holloway, & Begun, 2011; Oakeshott et al., 1983; Sezgin et al., 2004), suggesting that convergent evolution in similar environments may have shaped variation in these traits, albeit in different parts of the world. The expansion to northern habitats is suggested to be a relatively recent phenomenon in the history of *D. melanogaster*. This species is thought to have migrated from equatorial zones to northern latitudes 10,000–20,000 years ago (Begun & Aquadro, 1993; David & Capy, 1988). Migration to certain geographic areas, such as North America and Australia, is thought to be much more recent, and as late as the last 200 years (Hoffmann & Weeks, 2007; Keller, 2007; Knibb, 1982). While the recent migration into these areas may be indicative of a population bottleneck, thereby reducing genetic heterogeneity, gene expression analyses of low and high latitude North American populations revealed significant differences in gene expression between these populations (Svetec et al., 2015; Zhao, Wit, Svetec, & Begun, 2015), suggesting that these populations are indeed genetically dissimilar. It is possible that reduced sleep is associated with this migration and is an evolutionary adaptation to seasonal changes in temperature and light cycle (Adrion et al., 2015; Li & Stephan, 2006). Achieving a better understanding of the evolution and biogeography of *D. melanogaster* in the geographic regions investigated in this study will help inform the relationship between sleep and the metabolic traits measured here. Overall, our results reveal dramatic variation in sleep and metabolic regulation. Although our findings revealed that the associations between sleep and metabolic traits with the geographic variables examined were moderate, our observations associating clinal variation with naturally occurring differences in sleep‐related traits suggests that environmental gradients are potent selective forces that can shape behavior. In addition to the variables examined here, there are numerous additional factors that have been previously shown to influence sleep behavior, including lighting (Menegazzi et al., 2017), daily changes in temperature (Parisky et al., 2016), and composition of food resources (Catterson et al., 2010). Future studies examining the response of these and similar populations to changes in these environmental factors, and how they associate with sleep and metabolic function, may shed light on the environmental factors that modulate variation in sleep regulation.

## CONFLICT OF INTEREST

The authors declare no conflicts of interest.

## AUTHOR CONTRIBUTIONS

A.C.K. and E.B.B. designed the study and wrote the manuscript. E.B.B., J.T., V.R., and A.K. conducted the sleep and starvation experiments. R.A.B. and J.R.D. performed the nutrient storage measurements. E.B.B. analyzed the data. All authors read and approved of the manuscript.
