# Supplementary figures and images for: Variation in sleep and metabolic function is associated with latitude and average temperature in Drosophila melanogaster
Source: Ecol Evol. 2018 Mar 26;8(8):4084–97. doi: 10.1002/ece3.3963 (PMC5916307; doi:10.1002/ece3.3963)

Figure S1

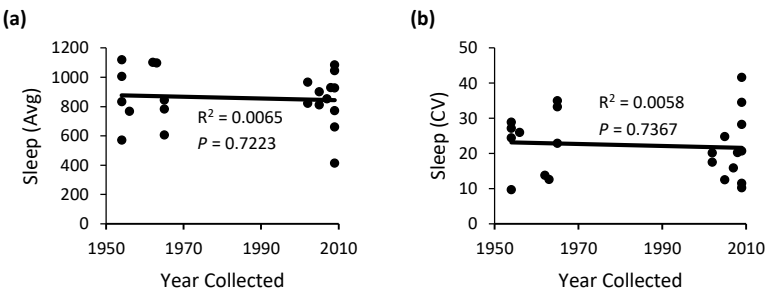

Supplement: Supplementary file 1 [file ECE3-8-4084-s001.pdf]
